# P03-016 - ANTI IL1 refractory CINCA respondes to ANTI IL6

**DOI:** 10.1186/1546-0096-11-S1-A214

**Published:** 2013-11-08

**Authors:** G Dueckers, K Sinha, V Soditt, G Ganser, T Niehues

**Affiliations:** 1 Helios Kliniken Krefeld, Krefeld, Germany; 2 Helios Kliniken Wuppertal, Wuppertal, Germany; 3 Klinikum Solingen, Solingen, Germany; 4 St. Josef Stift, Kinderrheumatologie, Sendenhorst, Germany

## Introduction

CINCA is thought to be mainly due to a dysregulation of IL1 synthesis and many patients respond to IL1 inhibition. The role of other cytokines in CINCA disease pathogenesis is less clear.

## Case Report

A 13 year old boy from a healthy non-consanguineous family suffers from severe autoinflammatory symptoms (persistent fever, polyserositis, urticaria, arthritis, developmental delay, deafness and hepatosplenomegaly) since his first year of life. Based on clinical presentation and genotype (heterozygous mutation (CAG)>Lysin (AAG)-p.Gln703Lys/Q705K substitution in Exon 3 of NLPR3) he was diagnosed for CINCA, an IL-1 driven cryopyrinopathy. Despite intensive pretreatment (NSAI, GC pulses, MTX, Anakinra and Canakinumab), no durable control of autoinflammation was achievable. With the initiation of Tozilizumab, an IL-6 receptor antagonist, all signs of autoinflammation disappeared, ESR and Serum Amyloid normalized within days. Finally the boy was even able to build a snowman without any joint stiffness or arthralgias for the first time of his life. The patient is now in stable clinical remission for > 6 months.

## Discussion

This is the first report of a patient with CINCA, who achieved clinical remission with the inhibition of IL6 pathway for more than 6 months. If Canakinumab fails to control autoinflammation in CINCA inhibition of IL-6 pathway might be an alternative. The mechanism of action of Tozilizumab in cryopyrinopathies remains to be elucidated.

## Competing interests

None Declared.

